# Nanophase diagram of binary eutectic Au-Ge nanoalloys for vapor-liquid-solid semiconductor nanowires growth

**DOI:** 10.1038/srep11263

**Published:** 2015-06-08

**Authors:** Haiming Lu, Xiangkang Meng

**Affiliations:** 1 National Laboratory of Solid State Microstructures, Collaborative Innovation Center of Advanced Microstructures, College of Engineering and Applied Sciences, Institute of Materials Engineering, Nanjing University, Jiangsu 210093, People’s Republic of China

## Abstract

Although the vapor-liquid-solid growth of semiconductor nanowire is a non-equilibrium process, the equilibrium phase diagram of binary alloy provides important guidance on the growth conditions, such as the temperature and the equilibrium composition of the alloy. Given the small dimensions of the alloy seeds and the nanowires, the known phase diagram of bulk binary alloy cannot be expected to accurately predict the behavior of the nanowire growth. Here, we developed a unified model to describe the size- and dimensionality-dependent equilibrium phase diagram of Au-Ge binary eutectic nanoalloys based on the size-dependent cohesive energy model. It is found that the liquidus curves reduce and shift leftward with decreasing size and dimensionality. Moreover, the effects of size and dimensionality on the eutectic composition are small and negligible when both components in binary eutectic alloys have the same dimensionality. However, when two components have different dimensionality (e.g. Au nanoparticle-Ge nanowire usually used in the semiconductor nanowires growth), the eutectic composition reduces with decreasing size.

One-dimensional structures such as nanotubes and nanowires are being investigated for various applications in nanotechnology. In particular, Ge and Si nanowires have been extensively studied due to their unique properties and potential applications in nanoelectronics^1^, light emitters and detectors^2^, photovoltaics^3^ and thermoelectrics^4^. A common technique for growing these semiconductor nanowires is the vapor-liquid-solid (VLS) mechanism originally suggested by Wagner and Ellis^5^. In the VLS growth process, gas precursors containing reactant material catalytically decompose and then the elements to be deposited dissolve into the catalyst to form a molten alloy droplet with the catalyst. This liquid droplet continues to adsorb decomposing solute atoms from the vapor, leading to a supersaturated state at which time crystallization of the semiconductor occurs at the liquid-solid interface resulting in nanowires growth^6^. This growth technique has been studied for a number of years, but it is not yet completely understood^7^.

Although the nanowire growth is a non-equilibrium process, the equilibrium phase diagram of binary alloy provides important guidance on the growth conditions, such as the temperature and the equilibrium composition of the alloy. For a fixed amount of metal, the equilibrium composition affects some important aspects of the growth, such as the alloy drop size, the supersaturation and the growth rate^8^,^9^. Given the small dimensions of the VLS alloy seeds (few tens of nanometers) and the nanowires, the known phase diagram of the corresponding bulk binary alloy cannot be expected to accurately predict the behavior of the nanowire growth. Indeed, there are often discrepancies between the actual growth results and the description based on the bulk phase diagram^10^.

There are some experimental and theoretical progresses in understanding how the phase diagram of alloys changes with decreasing system size^7^,^8^,^9^,^11^,^12^,^13^,^14^,^15^,^16^,^17^,^18^,^19^. For example, it has been suggested that capillary effects, often represented by the Gibbs-Thomson pressure, can account for the change of phase diagram^12^,^16^. Adhikari *et al.* calculated the Gibbs-Thomson modified equilibrium phase diagram of Au-Ge in the nanometer-scale regime and found that the growth-geometry eutectic temperatures were lower by 42, 22 and 11 K for nanowires with the diameter of 10, 20 and 40 nm than the corresponding bulk value^16^. In this method, the size effect is realized through separating the thermodynamic quantities into bulk and surface items, related to the contribution of the surface/volume ratio. However, as the size of the nanophase decreases to the size comparable with the atomic diameter (i.e. several nanometers), the size effect becomes stronger than the surface/volume ratio^20^. Different from the above conclusion of only small deviations from the bulk phase behavior, Schwalbach and Voorhees predicted a significant size-dependent phase diagram of Au-Ge nanoalloy through considering the effects of surface energy and surface stress to the diffusion and chemical potentials for the various phases in the nanoscale system, where the liquidus for the 30 nm diameter nanowires is about 40 K below the bulk value and more than 100 K below for the 10 nm diameter nanowires^7^. However, their calculated Au-Ge liquidus exhibits a roughly constant downward shift across a wide composition range that depends on the diameter of the nanowires, which is in disagreement with the experimental observation that the shift is relatively small near the eutectic composition and increases for higher Ge compositions in the liquid^8^. Moreover, the known studies of the nanophase diagram have neglected the atomic interaction energy between different components and its size dependence^11^,^17^. Here, we calculated the nanophase diagrams of Au-Ge binary eutectic alloys based on the size-dependent cohesive energy model^21^.

## Formula

According to the thermodynamic theory of phase diagram, the thermodynamic equilibrium between the solid and the liquid occurs when the chemical potentials *μ* of component *i* (*i* may be component *A* or *B*) in each phase is the same, namely


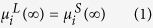


where the superscripts *L* and *S* respectively denote the liquid and the solid, and ∞ denotes the bulk.

In terms of the bulk phase diagrams of Au-Ge, the maximal solid solubility between Ge and Au is only 0.4% (atom percentage)^22^. Since the solid solubility is so small and the solidus lines almost have a negligible effect on the VLS growth process, the solid solubility is thus assumed to be zero as a first-order approximation to simplify the derivation and thus the solidus lines will not occur in our calculated phase diagram. In this case,


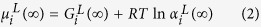



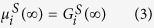


where *G* is the molar Gibbs free energy, *R* is the ideal gas constant, and *T* is the temperature. *α* = *ηx* is the activity with activity factor *η* and atomic percentage *x*. For a binary regular solution, ln*η*_*i*_ = (Ω^*L-i*^/*RT*)(1-*x*_*i*_)^2^ in a quasichemical approach with Ω being the atomic interaction energy^13^. According to the bulk phase diagrams of binary eutectic alloy, *i* is component *A* at *x*_*B*_ below the bulk eutectic composition *x*_*e*_ while *i* is component *B* at *x*_*B*_ ≥ *x*_*e*_.

It is known that 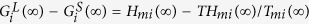 where *H*_*m*_ and *T*_*m*_ denote the melting enthalpy and the melting temperature of component *i*, respectively. Substituting Eqs. (2) and (3) and the above considerations into Eq. (1), we can obtain


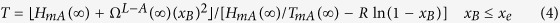



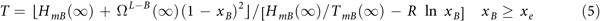


**Figure 1:**
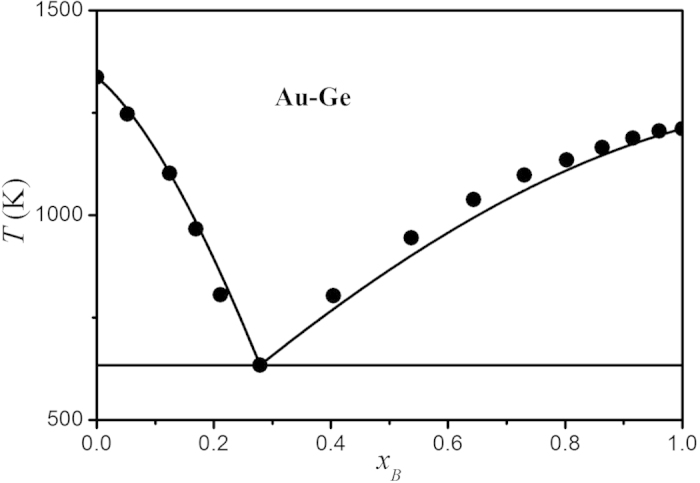
Comparison of Au-Ge bulk equilibrium phase diagrams between the model predictions based on Eqs. (4) and (5) (the solid lines) and the experimental results (the closed circles)^22^.

When *x*_*B*_ is certain, *T* is unique in the bulk binary eutectic phase diagram and can be determined based on Eqs. (4) and (5) when other parameters are known, noted that *T* is equal to the bulk eutectic temperature *T*_*e*_(∞) at *x*_*B*_ = *x*_*e*_. Eqs. (4) and (5) can also be employed to determine Ω^*L-i*^(∞) when other parameters are known. Considering the fact that the electronegativity difference between Au and Ge is small and comparable with that in the continuous solution phase diagram (e.g. Au-Ag), Ω^*L-i*^(∞) can be assumed to be weak function of the composition. Thus, through taking *x*_*e*_ and *T*_*e*_(∞) from the bulk phase diagram^22^ and the known values of *H*_*mi*_(∞) and *T*_*mi*_(∞) from reference 23, the bulk atomic interactive energies Ω^*L-i*^(∞) for Au-Ge alloys can be determined based on Eqs. (4) or (5) and are show in Table 1. Further, together with the calculated Ω^*L-i*^(∞) values, the bulk equilibrium phase diagram of Au-Ge alloy can be plotted in terms of Eq. (4) and (5) and shown in Fig. 1, where the experimental results taken from reference 22 are also listed for a comparison. It can be found that the model predictions agree with the corresponding experimental results, which in return confirms the validity of the fitting Ω^*L-i*^(∞) values and enables us to determine the nanophase diagram.

Since Au-Ge nanoalloys have the same structure as the corresponding bulk crystals, the above deductions and expressions may be extended to the nanometer size. Thus, the size- and composition-dependent liquidus temperature can be described as


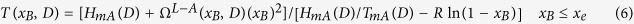



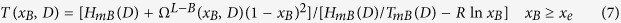


where *D* is the diameter of nanoparticles and nanowires. Combining Eq. (6) with Eq. (7), we can determine the eutectic composition and eutectic temperature at certain size.

The melting enthalpy *H*_*m*_ and melting temperature *T*_*m*_ are proportional to the bond energy and have been deduced to have the same size dependences as the cohesive energy^21^, namely


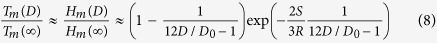


where *S* = *H*_*b*_(∞)/*T*_*b*_(∞) is the bulk solid-vapor transition entropy of crystals with *H*_*b*_(∞) and *T*_*b*_(∞) being the bulk enthalpy of vaporization and boiling temperature, respectively. *D*_0_ is a critical size at which all atoms of crystal are located on its surface, which can be determined as: (1) *D*_0_ = 6*h* for nanoparticles with dimensionality *d* = 0 since 4π(*D*_0_/2)^2^*h* = 4π(*D*_0_/2)^3^/3 where *h* is the atomic diameter; (2) *D*_0_ = 4*h* for nanowires with *d* = 1 since 2π(*D*_0_/2)*h* = π(*D*_0_/2)^2^; and (3) *D*_0_ = 2*h* for thin films with *d* = 2. In short, the correlation between *D*_0_ and *h* is given by,


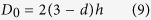


It is known that Ω = *ZN*_a_[*ε*_*AB*_-(*ε*_*AA*_ + *ε*_*BB*_)/2] where *Z*, *N*_a_ and *ε* denote the coordination number, Avogadro′s constant and the bond energy. Since the atomic interaction energy is also proportional to the bond energy, its size dependence should also be the same as that of the cohesive energy. Combining Eq. (8) with the consideration of the composition effect, a unified model to describe size- and composition-dependent atomic interaction energy Ω(*x*,*D*) can be developed as


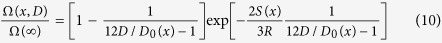


As a first-order approximation, the Fox equation can be used to calculate the composition-dependent bulk vaporization entropy *S*(*x*) and critical size *D*_0_(*x*) as following^24^


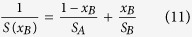



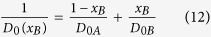


Combining Eqs. (8)-(12), 10, 14, 14, 14 into Eqs. (6) and (7), binary eutectic nanophase diagram can be determined.

## Results

**Figure 2:**
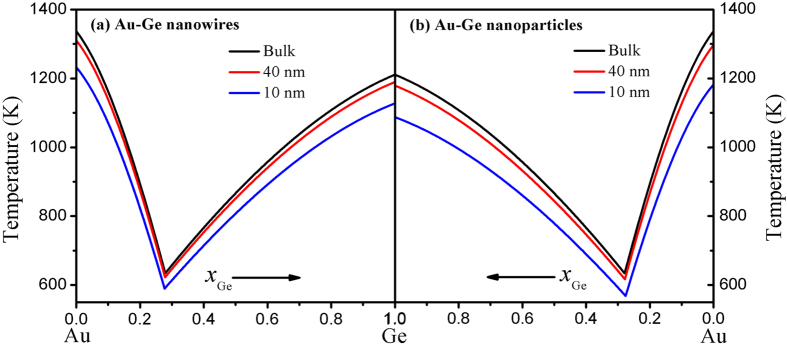
Equilibrium phase diagrams of binary eutectic alloy plotted based on Eqs. (6) and (7) for (**a**) Au-Ge nanowires and (**b**) Au-Ge nanoparticles.

Figure. 2a shows the calculated equilibrium phase diagram of Au nanowires-Ge nanowires in terms of Eqs. (6) and (7) with the diameters of 40 and 10 nm, where the calculated bulk liquidus curves of Au-Ge are also presented for a comparison. It can be found the liquidus curves drop as the diameter decreases and the downshift across a wide composition range is not constant but relatively small near the eutectic composition and then increases for higher Ge or Au compositions in the liquid. For example, the eutectic temperature of 10 nm nanowires is lowered by 43 K in comparison with the bulk value while the melting temperatures of pure Au and Ge are 105 and 83 K lower than the corresponding bulk melting temperatures. The downward trend of liquidus curves with composition is also in agreement with the experimental observations^8^,^9^. Besides the depression of the eutectic temperature, the eutectic composition is also found to slightly decrease from 0.279 to 0.277 when the diameter of nanowires reduces from the bulk to 10 nm. Similar effects are also visible for the Au-Ge binary eutectic nanoparticles shown in Fig. 2b while the depression extent is larger than that in nanowires. For instance, the eutectic temperatures of 40 and 10 nm nanoparticles are 617 and 568 K, which are 6 and 22 K lower than those of the nanowires with the same diameters, respectively. However, the eutectic compositions of nanoparticles with the diameters of 40 and 10 nm are determined to be 0.2781 and 0.276, which are closed to 0.2785 and 0.277 of the corresponding nanowires. These indicate that both the diameter and the dimensionality have effects on the nanophase diagram where the effects on the eutectic temperature are obvious while those on the eutectic composition are small and negligible as a first-order approximation. Because the data of equilibrium nanophase diagram of Au-Ge nanowires and nanoparticles are unavailable for us, no comparison is listed in Fig. 2.

**Figure 3:**
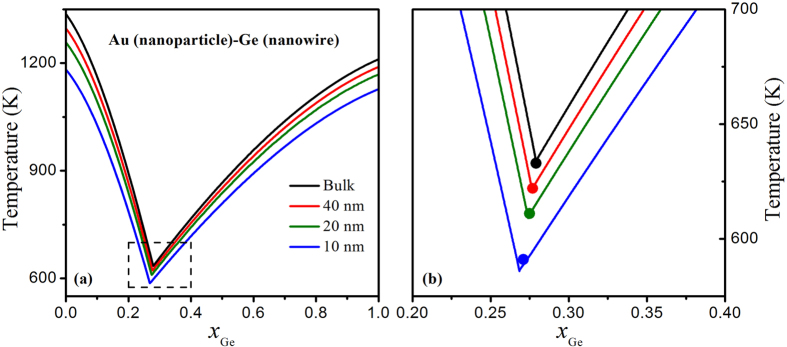
(**a**) Equilibrium phase diagrams of bulk Au-Ge and Au nanoparticle-Ge nanowire plotted based on Eqs. (4)–(7). (b)Eqs. (4)–(7). (b)Eqs. (4)–(7). (b)Eqs. (4)–(7). (b) Detail of the region represented by the squares in Fig. 3a where experimental (the black closed circle) and other theoretical predictions (other solid circles) on the eutectic temperature and composition are also shown^16^.

In Fig. 2, both Au and Ge have the same dimensionality *d* in each phase diagram, i. e. *d* = 1 for Fig. 2a or *d* = 0 for Fig. 2b. However, in the VLS semiconductor nanowires growth, Au is nanoparticle while Ge is semiconductor nanowire and then they have different dimensionality. Figure 3a shows the calculated equilibrium phase diagrams of Au nanoparticle-Ge nanowire in terms of Eqs. (6) and (7) with the diameters of 40, 20 and 10 nm, noted that the calculated bulk diagram phase of Au-Ge are also presented for a comparison. It can be found the liquidus curves also decrease with the decreasing diameter, similar to that in Fig. 2. Figure 3b is the detail of the region near the eutectic composition represented by the square in Fig. 3a. It is obvious that both the eutectic composition and the eutectic temperature reduce with the decreasing diameter. Based on Eqs. (4)-(7), 10, 10, 10, the eutectic temperatures and compositions of Au nanoparticles-Ge nanowires are determined to be 634 K and 0.279 for the bulk, 622 K and 0.276 for the 40 nm diameter, 609 K and 0.274 for the 20 nm diameter, 586 K and 0.269 for the 10 nm diameter, which are in agreements with experimental results of 633 K and 0.279 for the bulk^22^, and other theoretical predictions of 622 K and 0.277 for the 40 nm diameter, 611 K and 0.275 for the 20 nm diameter, 591 K and 0.271 for the 10 nm diameter^16^. As shown in Fig. 3a, the downward of liquidus curves is also relatively small near the eutectic composition and then increases for higher Ge compositions in the liquid while the model proposed by Adhikari *et al.* give the opposite prediction as shown in Fig. 3a of reference 16, which is contrary to the experimental observations^8^,^9^.

## Discussions

Based on Eq. (6), the eutectic temperatures and compositions of Au-Ge are determined to be 590 K and 0.277 for the 10 nm diameter nanowires, 568 K and 0.276 for the 10 nm diameter nanoparticles, and 586 K and 0.269 for the 10 nm diameter Au nanoparticles-Ge nanowires. It is evident that the eutectic temperature of Au nanoparticles-Ge nanowires is in-between Au-Ge nanowires and Au-Ge nanoparticles while its eutectic composition is smaller than those of other two systems. It is understandable because only the size effect exists in Au-Ge nanowires or nanoparticles systems and then the downward trends of both liquidus curves are comparative, which results in a small change of the eutectic composition. However, in Au nanoparticles-Ge nanowires system, the depression extent of the left liquidus curve is larger than that of the right liquidus curve, which leads to a large change of the eutectic composition. Thus, it can be concluded that the effects of size and dimensionality on the eutectic temperature and the eutectic composition are evident and not negligible in Au nanoparticles-Ge nanowires, which is different from those in Au-Ge nanowires or nanoparticles.

**Figure 4:**
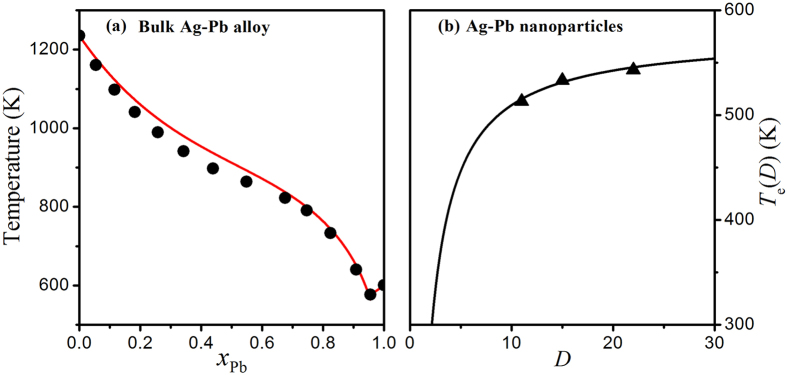
(**a**) Equilibrium phase diagram of bulk Ag-Pb eutectic alloy described by Eqs. (4) and (5) where the symbols (closed circles) are the corresponding experimental results^22^. (**b**) Comparison of *T*_*e*_(***D***) function of Ag-Pb nanoparticles described by Eq. (6) and the corresponding experimental results^19^.

Our model can be applicable for other eutectic alloys where the solid solubility of both components is also small and negligible, e.g. Au-Si or Ag-Pb. Figure 4a shows the calculated equilibrium phase diagram of bulk Ag-Pb eutectic alloy described by Eqs. (4) and (5) where the parameters in equation are listed in Table 1. For a comparison, the corresponding experimental results taken from reference 22 are also shown in Fig. 4a, and the agreement between the model predictions and the corresponding experimental results can be found. This correspondence indicates that our model can satisfactorily depict the equilibrium phase diagram of bulk Ag-Pb eutectic alloy. Recently, Chen *et al.* have investigated the eutectic transition of Ag-Pb alloy nanoparticles using *in situ* transmission electron microscopy and found that the eutectic temperature also decreased with the decreasing particle size^19^. According to the above results and discussions, the eutectic composition of binary system is found to slightly shift and the change is negligible as a first-order approximation when both components have the same dimensionality. Under this assumption, Fig. 4b compares the size-dependent eutectic temperature *T*_*e*_(*D*) of Ag-Pb alloy nanoparticles between the model predictions based on Eq. (6) with *D*_0_ = 6*h* and the corresponding experimental observations^19^. It can be found that the predicted 546, 531 and 516 K for the nanoparticles with the diameters of 22, 15 and 11 nm are in good agreements with the experimental observations of 543, 533, and 513 K^19^.

It is known that exp(-*x*) ≈ 1-*x* when *x* is small enough, e.g. *x* < 0.1. Together with *H*_*m*_(*D*)/*T*_*m*_(*D*) ≈ *H*_*m*_(∞)/*T*_*m*_(∞) based on Eq. (8), Eq. (6) can be rewritten as (the same approximation can also be made to Eq. (7)


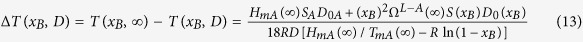


It is obvious according to Eq. (13) that there is a linear relationship between Δ*T*(*x*_*B*_,*D*) or *T*(*x*_*B*_,*D*) and 1/*D* at a certain *x*_*B*_ when *D* is large enough (e.g. *D* > 10 nm). Since the eutectic temperature locates in the liquidus curves, the conclusion^19^ that a linear relationship was obtained for the eutectic temperature as a function of the reciprocal of the particle size is understandable and inevitable in this case. Noted that *D*_0_ = 6*h* and 4*h* for nanoparticle and nanowire, Δ*T*_nanoparticles_(*x*_*B*_,*D*)/Δ*T*_nanowires_(*x*_*B*_,*D*) = 3:2 according to Eq. (13) when *D* is large enough. However, as the size of the nanocrystals further decreases to the size comparable with the atomic diameter, namely several nanometers, the size effect of the thermodynamic parameters is stronger than Eq. (13) since the energetic state of interior atoms of the nanocrystals in a small size is higher than that of the corresponding bulk crystals^25^.

In the models proposed by Adhikari and Schwalbach *et al.*^7^,^16^, the solid-vapor surface energy was thought to the reason for the depression of the liquidus. Note that surface energy of the liquid and the crystal can be determined by the surface broken number and the cohesive energy^26^, where the surface broken number is related to the geometry and dimensionality of the crystals. Thus, it can be claimed that the validity of our model is based on the combination of size-dependent cohesive energy with the dimensionality of the crystals, and hence the effect of surface energy has been implied in our theoretical model. Moreover, as the size decreases to below about 10 nm, the size dependence of surface energy becomes evident^26^, while this size dependence was neglected in the models of Adhikari and Schwalbach *et al.*^7^,^16^.

Recently, Marshall *et al.* have found the formation of a hexagonal close-packed (hcp) structure in Au catalyst nanoparticles during crystallization following Ge nanowires growth^27^. This unusual observation of hcp Au was suggested to result from the ability of the Au-Ge system to form the structurally similar metastable hcp alloy β-phase^27^. The occurrence of hcp structure rather than equilibrium face-centered-cubic structure in Au will affect the values of thermodynamic parameters in Eqs. (4)-(7), 10, 10, 10 and finally alter the nanophase diagram of Au-Ge alloy.

## Additional Information

**How to cite this article**: Lu, H. M. & Meng, X. K. Nanophase diagram of binary eutectic Au-Ge nanoalloys for vapor-liquid-solid semiconductor nanowires growth. *Sci. Rep.* **5**, 11263; doi: 10.1038/srep11263 (2015).

## Figures and Tables

**Table 1:** Related parameters and data used in equations where *x*_*e*_ and *T*_*e*_ values are cited from reference^22^ while *h*, *T*_*m*_, *H*_*m*_, *T*_*b*_, and *H*_*b*_ values are taken from reference 23.

	***h***	***x*_*e*_**	***T*_*e*_**	***T*_*m*_**	***H*_*m*_**	***T*_*b*_**	***H*_*b*_**	***S* = *H*_*b*_/*T*_*b*_**	**Ω^*L-i*^**
	**(nm)**		**(K)**	**(K)**	**(kJ/mol)**	**(K)**	**(kJ/mol)**	**(J/mol-K)**	**(kJ/mol)**
Ge	0.245	0.279	633	1211.4	31.8	3093	334	108.0	−16.2
Au	0.288			1337	12.5	3129	330	105.5	−62.3
Ag	0.289			1235	11.3	2435	255	104.7	9.7
Pb	0.350	0.955	577	600.6	4.77	2022	178	88.0	15.0

Ω is determined through fitting Eq. (4) to the corresponding bulk phase diagram.
